# Kinder- und Jugendsport „nach“ Corona: Die Bedeutung von Bewegung, Spiel und Sport für ein gesundes Aufwachsen

**DOI:** 10.1007/s43594-022-00057-w

**Published:** 2022-03-25

**Authors:** Nils Neuber

**Affiliations:** Institut für Sportwissenschaft, Westfälische Wilhelms-Universität Münster, Münster, Deutschland

Unter dem Titel „Tickende Zeitbombe“ schrieb der *Spiegel* am 17. April 2021: „Eine aktuelle Studie ermittelt drastische Bewegungseinbrüche bei Kindern und Jugendlichen in der Pandemie. Die körperlichen Folgen könnten lebenslang anhalten, sagen Experten und warnen vor einer ‚verlorenen Generation‘“ (Windmann 2021, S. 90). Hintergrund des Artikels war die Veröffentlichung einer Auswertung der Motorik-Modul-Studie (MoMo) des Karlsruher Instituts für Technologie (KIT) zur körperlich-sportlichen Aktivität von Kindern und Jugendlichen während der Covid-19 Pandemie in Deutschland (Schmidt et al. 2021). Sportaktivitäten junger Menschen haben danach in den ersten beiden Corona-Wellen deutlich abgenommen (Abb. 1), während die Bildschirmnutzung in der Freizeit deutlich zugenommen hat. Aber kann man deshalb schon von einer „verlorenen Generation“ sprechen? Sind die Folgen wirklich nicht mehr aufzuholen? Um welche Folgen geht es überhaupt konkret? Und wie müsste sich der Kinder- und Jugendsport entwickeln, um ein gesundes Aufwachsen von Kindern und Jugendlichen „nach“ Corona sicherzustellen?

**Figure 1:**
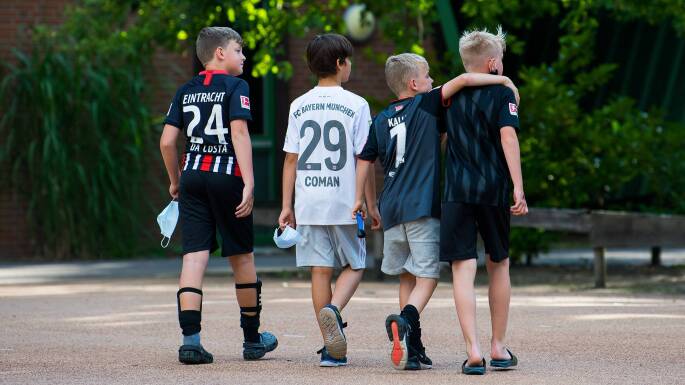

## Bedeutung von Bewegung, Spiel und Sport für das Aufwachsen von Kindern und Jugendlichen

**Figure 2:**
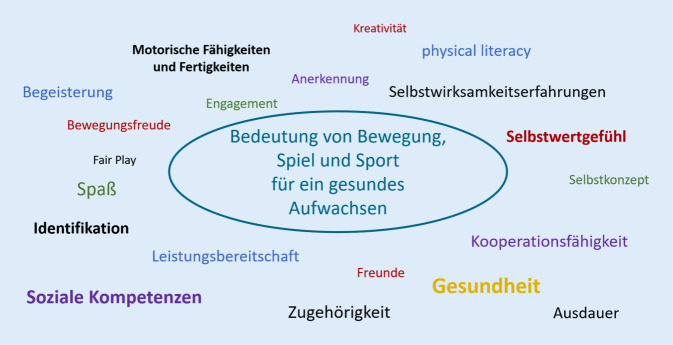

Auf den ersten Blick sind Bewegungs- und Sportaktivitäten eng mit der Entwicklung motorischer Fähigkeiten und Fertigkeiten verbunden. Die motorische Leistungsfähigkeit gilt als wichtiger Marker für die Gesundheit von Heranwachsenden (Klemm 2021). Auf den zweiten Blick rücken zahlreiche weitere Aspekte in den Fokus, wenn die Bedeutung von Bewegung, Spiel und Sport für ein gesundes Aufwachsen diskutiert wird: Leistungsbereitschaft, Selbstkonzept und Kooperationsfähigkeit zum Beispiel oder auch Bewegungsfreude, Anerkennung und Freundschaft (Abb. 2). Tatsächlich scheint das Spektrum der Bewegungsbedeutungen deutlich größer zu sein, als es der Spiegel-Artikel mit seinen „lebenslangen körperlichen Folgen“ suggeriert. In einem Kommentar für das *Forum Kinder- und Jugendsport* hat Martin Schönwandt die Engführung der Diskussion in Bezug auf die WHO-Empfehlungen zur täglichen Bewegungszeit von Kindern und Jugendlichen (60 min täglich) kritisiert: Das Erleben junger Menschen lässt sich „nicht nur in Zahlen ausdrücken […], sondern auch in Interaktion, Partizipation, Natur- und Körpererleben, Begeisterung, Freundschaften und vielem mehr“ (Schönwandt 2021, S. 97).

**Figure 3:**
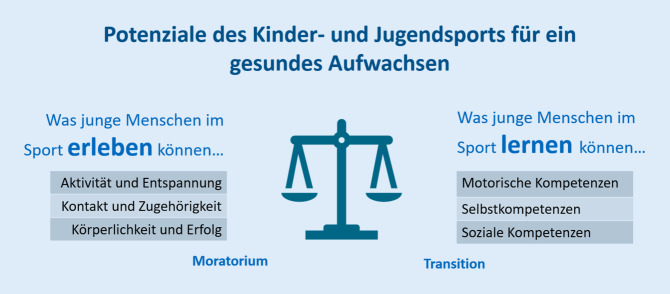

Genau diese Vielfalt an Bedürfnissen und Wirkungen scheint mir der Schlüssel zum Verständnis der Bedeutung von Bewegung für das Aufwachsen junger Menschen zu sein, auch und gerade mit Blick auf ihre Gesundheit. Zieht man nämlich gesundheitswissenschaftliche Verständnisweisen hinzu, ist Gesundheit weit mehr als ein funktionelles Optimum oder das „Schweigen der Organe“. Vielmehr wird Gesundheit heute als ein Gleichgewicht des subjektiven und objektiven Befindens einer Person verstanden, das dann gegeben ist, „wenn diese Person sich in den physischen, psychischen und sozialen Bereichen ihrer Entwicklung im Einklang mit den eigenen Möglichkeiten und Zielvorstellungen und den jeweils gegebenen äußeren Lebensbedingungen befindet“ (Hurrelmann, 2010, zit. nach Erlemeyer 2016, S. 19). Es geht also um subjektive Erlebniskategorien, wie Aktivität und Entspannung, Kontakt und Zugehörigkeit oder Körperlichkeit und Erfolg, und *zugleich* um objektive Lernkategorien wie motorische Kompetenzen, Selbstkompetenzen oder soziale Kompetenzen (Abb. 3). Zur Darstellung dieser Perspektivenvielfalt kann das Modell der Zeitperspektiven von gegenwartsbezogenen Entfaltungsbedürfnissen (Moratorium) und zukunftsbezogenen Entwicklungsaufgaben (Transition) aus der Jugendforschung genutzt werden (Neuber et al. 2021).

## Beeinträchtigungen durch die Pandemie

Schaut man aus dieser Perspektive auf die Beeinträchtigungen von Kindern und Jugendlichen während der Corona-Pandemie, ergibt sich ein komplexes Bild, das motorische Defizite nicht ausblendet, letztlich aber deutlich darüber hinausweist. Im Folgenden werden einige ausgewählte Befunde für Deutschland vorgestellt, wobei die Situation im europäischen Ausland ähnlich aussieht (Naul 2021). So zeigt sich im Hinblick auf den Bereich *Alltagsleben und Medienkonsum,* dass Kinder- und Jugendliche in Deutschland während der ersten Welle der Pandemie täglich deutlich weniger Zeit mit Schulbesuch und schulischen Aufgaben verbracht haben (minus 3,8 h). Stattdessen nahmen mediale Aktivitäten (Fernsehen, Computer- oder Handyspiele, Nutzung sozialer und Online-Medien) um 1,3 h täglich zu. Die Zeit für kreative Tätigkeiten, Lesen und Bewegung stieg mit 0,3 h pro Tag nur leicht. Während die Zeit für Schulaktivitäten bei Kindern von Akademiker*innen in etwa gleichem Maße gesunken ist wie bei denen von Nicht-Akademiker*innen, verbrachten Kinder von Akademiker*innen etwas mehr Zeit mit Lesen, kreativen Tätigkeiten und Bewegung als diejenigen von Nicht-Akademiker*innen und etwas weniger Zeit mit Medien (Wößmann et al. 2020).

In einer Studie von Langmeyer et al. (2020) zeigte sich, dass bei Kindern im Kindergartenalter die Nutzung von Medien wie Fernsehen, Streamingdiensten und YouTube (68 %) oder Radiohören (60 %) zugenommen hat, während die Zunahme bei digitalen Medien wie Spielen am Computer, Tablet oder Handy oder beim Surfen im Internet vergleichsweise konstant geblieben ist. Bei rund 40 % der Kinder nahm das Spielen draußen zu, bei 30 % ist dies weniger geworden. Mit steigendem Alter nahm allerdings die Zeit deutlich zu, die Heranwachsende mit „Nichts tun“, Computer spielen oder dem Surfen im Internet verbringen. Die Digitalisierung des Alltags hat zunehmend auch Auswirkungen auf das Körper- und Sportverständnis junger Menschen (Forschungsverbund Kinder- und Jugendsport NRW 2021a). Heranwachsende aus finanziell besser gestellten Familien spielen eher draußen, lesen eher Bücher oder bekommen sie vorgelesen und hören mehr Musik. Kinder aus finanziell schlechter gestellten Familien schauen mehr fern und spielen mehr am Computer/Handy. Die Zunahme digitaler Mediennutzung wird durch Daten der MoMo-Studie bestätigt. Dort wurde eine Zunahme der Bildschirmnutzungszeiten von durchschnittlich 130,9 min pro Tag vor der Pandemie über 191,2 min in der ersten Welle und 227,5 min in der zweiten Welle festgestellt (Schmidt et al. 2021, S. 13).

Zum Bereich der *Bewegungs- und Sportaktivitäten* von 4‑ bis 17-Jährigen stellen Schmidt et al. (2020 und 2021) fest, dass über alle Altersgruppen und beide Geschlechter hinweg die Zeit für sportliche Aktivitäten abgenommen hat (von minus 1,7 beziehungsweise 2,7 min täglich bei 4‑ bis 5‑jährigen Mädchen beziehungsweise Jungen bis hin zu minus 21,1 min bei 14- bis 17-jährigen Jungen). Dagegen hat die allgemeine körperliche Aktivität in der ersten Welle zunächst zugenommen. Am meisten gestiegen ist sie bei den 4‑ bis 5‑Jährigen (plus 58 min), während sich männliche Jugendliche nur zwischen 10 (14 bis 17 Jahre) und 16 min (11 bis 13 Jahre) mehr im Alltag bewegten. Die gleichaltrigen Mädchen bewegten sich mit etwa 22 bis 23 min etwas mehr. Allerdings sind diese Alltagsaktivitäten in der zweiten Welle – im Winter – deutlich unter den Ausgangswert vor der Pandemie zurückgegangen (Schmidt et al. 2021). Auch die Zeit für informelles Sporttreiben von älteren Jugendlichen und jungen Erwachsenen erhöhte sich zunächst um 17,7 min pro Tag. Nur rund 35 % der Befragten 14- bis 29-Jährigen reduzierten ihre Bewegungsaktivität in der Freizeit, fast 55 % behielten ihren Umfang wöchentlicher Aktivität bei und etwa 10 % erhöhten den Umfang (Mutz und Gerke 2020).

Die Kontaktbeschränkungen durch die Pandemie wirkten sich auch auf *Gesundheit und Wohlbefinden *von Kindern und Jugendlichen aus. In einer Befragung von Eltern 7‑ bis 10-jähriger Kinder sowie von 11- bis 17-jährigen Kindern und Jugendlichen in Deutschland fühlten sich 71 % der Befragten durch die Kontaktbeschränkungen belastet. 40 % berichteten von einer verminderten Lebensqualität im Vergleich zu 15 % vor der Pandemie (Ravens-Sieberer et al. 2020). In der zweiten Welle sind diese Werte noch einmal angestiegen (Ravens-Sieberer et al. 2021). Zusätzlich belasteten die Kontaktbeschränkungen sowohl die Beziehung zu Freunden als auch innerhalb der Familie (zum Beispiel mehr Streit innerhalb der Familie). Auch psychosomatische Beschwerden wie beispielsweise Kopfschmerzen, Gereiztheit oder Niedergeschlagenheit sind gegenüber der Zeit vor der Pandemie angestiegen. Hinzu kamen psychische Erkrankungen wie Depression oder Essstörungen, die deutlich zugenommen haben und nicht zuletzt auf fehlende Strukturen im Alltag zurückgeführt werden. Besonders betroffen sind sozial benachteiligte Kinder (Ravens-Sieberer et al. 2021). Dieser Befund deckt sich mit einer aktuellen Längsschnittstudie, in der motorische Leistungen und damit verbundene Gesundheitsparameter während der Corona-Pandemie signifikant mit der sozialen Herkunft korrelieren (Dreiskämper et al. 2021).

Massive Beeinträchtigungen zeigten sich auch im Bereich von *sozialer Teilhabe und Partizipation *junger Menschen. So äußern Jugendliche im Alter von 15 bis 30 Jahren den Eindruck, dass ihre Sorgen in der Pandemie nicht gehört wurden (23,6 %) und ihre Bedürfnisse nicht in den politischen Entscheidungen berücksichtigt wurden (Andresen et al. 2020). Sie monieren insbesondere die Schulzentrierung der Politik, die den Fokus ausschließlich auf schulisches Lernen richtet und Freiräumen im Aufwachsen junger Menschen wenig Beachtung schenkt. Ein junger Mensch sagt beispielsweise in der Befragung von Andresen et al. (2020, S. 4): „Wir Jugendlichen werden doch nur als Schüler gesehen. Wir sollen lernen und lernen und lernen. Warum wird darüber diskutiert, die Sommerferien zu kürzen. Politiker denken wie Kapitalisten“. Entsprechend fordert Voigts (2020, S. 98) mehr Freiräume für Jugendliche, auch in der Kinder- und Jugendarbeit im Sport: „Partizipation ermöglichen, Demokratieerleben ermöglichen, Bildung ermöglichen – und vor allem Freiräume, Beteiligungsräume und Bewegungsräume zu erkämpfen und für junge Menschen zur Verfügung zu stellen, ist dabei ein Schlüssel, um ihnen Unterstützung bei der Bewältigung ihrer Aufgaben anzubieten.“.

## Zusammenfassung der Befunde

Die Corona-Pandemie beeinträchtigt das Aufwachsen junger Menschen in vielfältiger Weise. Vorhandene Probleme und Herausforderungen treten in der Krise noch einmal stärker hervor und werden wie unter einem „Brennglas“ verdeutlicht (Leyendecker et al. 2020). Neben dem schulischen Lernen, das hier nicht näher beleuchtet wird, betrifft das insbesondere das *Erleben und Lernen in der Freizeit*. Die gemeinsamen Aktivitäten von Kindern und Jugendlichen sind ebenso beeinträchtigt wie das gemeinsame „Abhängen“, ihr Körperleben und ihre sportlichen Erfolge ebenso wie ihre sozialen Interaktionen und Freundschaften. Dennoch sind nicht alle Heranwachsenden gleichermaßen betroffen. Kinder und Jugendliche aus bildungsnahen Familien sind bislang vergleichsweise gut durch die Pandemie gekommen, während Heranwachsende aus *bildungsfernen Familien *besonders unter den Einschränkungen zu leiden hatten. Daher erscheint mir die Furcht vor einer „verlorenen Generation“ übertrieben, auch vor dem Hintergrund historischer Erfahrungen von Kriegsgenerationen (Jampert 1998). Gleichwohl gibt es nicht wenige junge Menschen, die jetzt dringend Unterstützung brauchen, um vorhandene Defizite aufholen zu können. Viele Aspekte sind bislang nur ansatzweise untersucht worden, vor allem die *Perspektive von Kindern und Jugendlichen *selbst fehlt weitgehend (Kauer-Berk et al. 2020). Trotzdem lassen sich die vorliegenden Befunde zum Kinder- und Jugendsport in der Corona-Pandemie im Sinne eines Zwischenfazits zusammenfassen

- Die Bedeutung von Bewegungs‑, Spiel- und Sportangeboten für das Aufwachsen von Kindern und Jugendlichen, insbesondere für ihre Entwicklung, ihre soziale Teilhabe und ihre Gesundheit, ist durch die Pandemie in besonderer Weise deutlich geworden – *qualitativ anspruchsvolle Bewegungs- und Sportaktivitäten sind mehr denn je entwicklungsbedeutsam!*
- Die Abhängigkeit der Bildungs- und Teilhabechancen junger Menschen von der sozialen Herkunft ist durch die Pandemie in besonderer Weise deutlich geworden – *sozial benachteiligte Heranwachsende werden in Bereichen wie Bildung, Teilhabe und Gesundheit besonders stark abgehängt!*
- Der veränderte Umgang mit dem eigenen Körper sowie die Entwicklung sozialer und gesundheitsbezogener Lebensstile ist unter den Bedingungen der Pandemie in besonderer Weise deutlich geworden – *Corona hat die Digitalisierung und „Entkörperlichung“ juveniler Lebensstile noch einmal beschleunigt!*

## Perspektiven für den Neustart

Ausgehend von dieser Zwischenbilanz lassen sich Perspektiven für einen Neustart des Kinder- und Jugendsports „nach“ Corona formulieren – womöglich auch „während“ oder „trotz“ Corona. Dazu hat es bereits einige Aufrufe gegeben. So hat der Forschungsverbund Kinder- und Jugendsport NRW (2021b) ein Positionspapier zu Bewegungs‑, Spiel- und Sportangeboten für Kinder- und Jugendliche nach der Corona-Pandemie veröffentlicht. Das Karlsruher Institut für Technologie formulierte einen Appell für einen Bewegungspakt (Woll et al. 2021). Der organisierte Kinder- und Jugendsport beteiligt sich am Aufholpaket des Bundes mit Bewegungs‑, Spiel- und Sportangeboten für Heranwachsende sowie einer Bewegungskampagne (zum Beispiel Deutsche Sportjugend 2021). Wichtig erscheint mir, die zahlreichen Aktivitäten und Initiativen zusammenfassend in den Blick zu nehmen. Ein systematischer Austausch von Erfahrungen und Befunden kann so zu einem differenzierten Verständnis für die Wechselwirkungen der für das* Aufwachsen junger Menschen* unter pandemischen Bedingungen relevanten Aspekte beitragen. Insofern begrüße ich die aktuelle Initiative der Sportministerkonferenz zur Vernetzung des Kinder- und Jugendsports durch die Einrichtung einer interdisziplinären Arbeitsgruppe mit Vertreter*innen aus dem Bildungs‑, Gesundheits‑, Sport- und Wissenschaftssystem ausdrücklich. Wichtig erscheint mir, dabei über Fragen der motorischen Beeinträchtigung hinauszugehen. Im Sinne des eingangs vorgestellten Modells der Zeitperspektiven lassen sich mindestens folgende Schwerpunkte herausstellen:

### Bewegung, Spiel und Sport erleben … (Moratorium)

**Figure 4:**
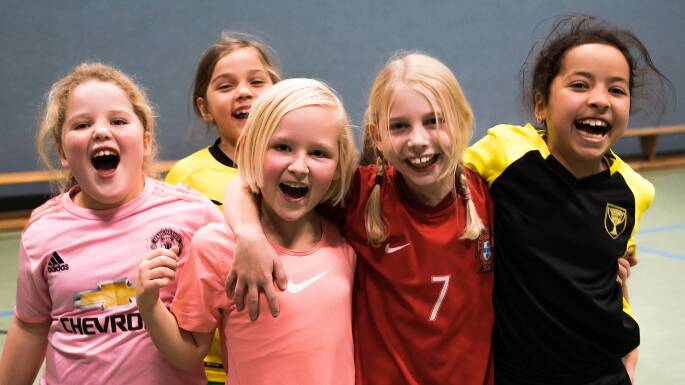

- Regelmäßige Bewegungs‑, Spiel- und Sportangebote in Kindertagesstätten und Schulen im Unterricht und in den Pausen.
- Förderliche Rahmenbedingungen für Bewegungs‑, Spiel- und Sportangebote in Sportvereinen und Kinder- und Jugendhilfe (Abb. 4).
- Öffnung kommunaler Räume wie Spielplätze, Schulhöfe und Sportanlagen für informelle Bewegungsaktivitäten.

### Mit Bewegung, Spiel und Sport lernen … (Transition)

- Förderung anspruchsvoller analoger und digitaler Bewegungsangebote für Kinder und Jugendliche, etwa zur motorischen Förderung, kognitiven Aktivierung oder sozialen Einbindung.
- Entwicklung neuer Angebote zum Umgang mit Körperlichkeit, Bewegung und Sport in digitalen Gesellschaften, auch zur kritischen Reflexion bestehender Praktiken.
- Integration sozial benachteiligter Kinder und Jugendlicher in den schulischen und außerschulischen Sport durch niederschwellige Angebotsformen, Sportpat*innenmodelle und kommunale Unterstützungsstrukturen.

### Bewegung, Spiel und Sport langfristig sichern … (politische Ebene)

- Vernetzung der Akteure des Kinder- und Jugendsports aus Praxis, Politik und Wissenschaft durch ein kontinuierliches Expert*innengremium auf Bundesebene, am besten durch ein langfristig angelegtes „Bundesjugendkuratorium Sport“.
- Systematische Förderung der Kinder- und Jugendsportforschung durch Einrichtung von BMBF-Förderlinien und Einbezug des Sports in die Kinder- und Jugendberichterstattung.
- Entwicklung einer Strategie zur Sicherung des Kinder- und Jugendsports unter den Bedingungen eines pandemischen Zeitalters nicht zuletzt auch mit Blick auf den Erhalt bürgerschaftlicher Strukturen.
